# Supplementary material for: Friendship paradox biases perceptions in directed networks
Source: Nat Commun. 2020 Feb 5;11:707. doi: 10.1038/s41467-020-14394-x (PMC7002371; doi:10.1038/s41467-020-14394-x)
Supplement: Supplementary file 1 — Supplementary Information [file 41467_2020_14394_MOESM1_ESM.pdf]

Supplementary Information

Friendship Paradox Biases Perceptions in Directed Networks

Nazanin Alipourfard*[1], Buddhika Nettasinghe*[2], Andrés Abeliuk[1], Vikram Krishnamurthy[2], and Kristina Lerman[1]

[1]Information Sciences Institute
[2]Cornell University

December 17, 2019

Contents

Supplementary Note 1: Derivations **2**
1.1 Derivation of Friendship Paradox Conditions 2
1.2 Derivation of B_{local} 3

Supplementary Note 2: Empirical Results **5**
2.1 Individual-level Perception Bias 5
2.2 Local and Global Bias 5
2.3 Global Bias Ranking 10

Supplementary Note 3: Polling Algorithm **10**
3.1 Follower Perception Polling (FPP) Algorithm 10
3.2 Bias of the Polling Estimate 10
3.3 Variance of the Polling Estimate 11
3.4 Heuristic Follower Perception Polling 12

* Alipourfard (nazanina@isi.edu) and Nettasinghe (dwn26@cornell.edu) contributed equally to this work.

Supplementary Note 1: Derivations

1.1 Derivation of Friendship Paradox Conditions

Theorem 1. *Let $G = (V, E)$ be a directed network. Then,*

1. *Random friend Y has more followers than a random node X, on average; i.e.,*

$$\mathbb{E}\{d_o(Y)\} - \bar{d} = \frac{\mathrm{Var}\{d_o(X)\}}{\bar{d}} \geq 0. \tag{1}$$

2. *Random follower Z has more friends than a random node X, on average; i.e.,*

$$\mathbb{E}\{d_i(Z)\} - \bar{d} = \frac{\mathrm{Var}\{d_i(X)\}}{\bar{d}} \geq 0. \tag{2}$$

Proof. Part 1:

$\mathbb{E}\{d_o(Y)\} - \mathbb{E}\{d_o(X)\} = \sum_{v \in V} d_o(v) \mathbb{P}(Y = v) - \sum_{v \in V} \frac{d_o(v)}{N}$

$$= \sum_{v \in V} d_o(v) \frac{d_o(v)}{\sum_{v' \in V} d_o(v')} - \frac{\sum_{v \in V} d_o(v)}{N} \tag{3}$$

$$= \frac{\frac{\sum_{v \in V} d_o(v)^2}{N} - \left(\frac{\sum_{v \in V} d_o(v)}{N} \right)^2}{\frac{\sum_{v' \in V} d_o(v')}{N}} \tag{4}$$

$$= \frac{\mathbb{E}\{d_o(X)^2\} - \mathbb{E}\{d_o(X)\}^2}{\mathbb{E}\{d_o(X)\}} = \frac{\mathrm{Var}\{d_o(X)\}}{\bar{d}} \geq 0 \tag{5}$$

Proof of part 2 follows using similar arguments. □

Theorem 2. *Let $G = (V, E)$ be a directed network where in-degree $d_i(X)$ and out-degree $d_o(X)$ of a random node X are positively correlated. Then,*

1. *Random friend Y has more friends than a random node X does, on average; i.e.,*

$$\mathbb{E}\{d_i(Y)\} - \bar{d} = \frac{\mathrm{Cov}\{d_i(X), d_o(X)\}}{\bar{d}} \geq 0. \tag{6}$$

2. *Random follower Z has more followers than a random node X does, on average; i.e.,*

$$\mathbb{E}\{d_o(Z)\} - \bar{d} = \frac{\mathrm{Cov}\{d_i(X), d_o(X)\}}{\bar{d}} \geq 0. \tag{7}$$

Proof. Part 1:

$\mathbb{E}\{d_i(Y)\} - \mathbb{E}\{d_i(X)\} = \sum_{v\in V} d_i(v)\mathbb{P}(Y=v) - \sum_{v\in V}\frac{d_i(v)}{N}$

$$= \sum_{v\in V} d_i(v)\frac{d_o(v)}{\sum_{v'\in V} d_o(v')} - \frac{\sum_{v\in V} d_i(v)}{N} \tag{8}$$

$$= \frac{\frac{\sum_{v\in V} d_i(v)d_o(v)}{N} - \left(\frac{\sum_{v\in V} d_i(v)}{N}\right)\left(\frac{\sum_{v'\in V} d_o(v')}{N}\right)}{\frac{\sum_{v'\in V} d_o(v')}{N}} \tag{9}$$

$$= \frac{\mathbb{E}(d_i(X)d_o(X)) - \mathbb{E}\{d_i(X)\}\mathbb{E}\{d_o(X)\}}{\mathbb{E}\{d_o(X)\}} = \frac{\mathrm{Cov}\{d_i(X), d_o(X)\}}{\bar{d}} \tag{10}$$

Hence, positive correlation ($\mathrm{Cov}\{d_i(X), d_o(X)\} > 0$) between in-degree $d_i(X)$ and out-degree $d_o(X)$ of a random individual X implies that $\mathbb{E}\{d_i(Y)\} > \mathbb{E}\{d_i(X)\}$.

Proof of part 2 follows using similar arguments. □

1.2 Derivation of B_{local}

Let Y' denote a uniformly sampled friend of a random node X. Further, let A_{uv} denote the element (u, v) of the adjacency matrix of network: $A_{uv} = 1$ if there is a link pointing from u to v and $A_{uv} = 0$ otherwise. Then, by definition of the function q_f in Section 2 of the main text,

$$q_f(X) = \frac{\sum_{U\in\mathcal{F}(X)} f(U)}{d_i(X)} = \mathbb{E}\{f(Y')|X\} \tag{11}$$

Therefore,

$$\mathbb{E}\{q_f(X)\} = \frac{1}{N}\sum_{v\in V}\left\{\frac{\sum_{u\in\mathcal{F}(v)} f(u)}{d_i(v)}\right\} = \frac{1}{N}\sum_{v\in V}\left\{\sum_{u\in v}\frac{f(u)}{d_i(v)}A_{uv}\right\} \tag{12}$$

$$= \frac{\sum_{u,v\in V} A_{uv}}{N}\sum_{v\in V}\left\{\sum_{u\in V}\frac{f(u)}{d_i(v)}\frac{A_{uv}}{\sum_{u,v\in V} A_{uv}}\right\} \tag{13}$$

$$= \bar{d}\times\mathbb{E}\left\{\frac{f(U)}{d_i(V)}\middle|(U,V)\sim\mathrm{Uniform}(E)\right\} \tag{14}$$

which yields the expression for the perception $\mathbb{E}\{f(X)\}$ of a random individual.

Next, assume, $f(U)$ and $\mathcal{A}(V)$ (where, (U, V) is a random link) are positively correlated ($\mathrm{Cov}\{f(U), \mathcal{A}(V)\} \geq 0$). Then,

$$\mathbb{E}\{q_f(X)\} = \bar{d}\mathbb{E}\left\{f(U)\mathcal{A}(V)\middle|(U,V)\sim\mathrm{Uniform}(E)\right\} \tag{15}$$

$$\geq \bar{d}\mathbb{E}\{f(U)|(U,V)\sim\mathrm{Uniform}(E)\} \tag{16}$$

$$\times\,\mathbb{E}\{\mathcal{A}(V)|(U,V)\sim\mathrm{Uniform}(E)\}$$

$$= \mathbb{E}\{f(Y)\} \tag{17}$$

Therefore, $\text{Cov}\{f(U), \mathcal{A}(V)\} \geq 0$ (condition (13) in the main text) is a necessary and a sufficient condition for $\mathbb{E}\{q_f(X)\} \geq \mathbb{E}\{f(Y)\}$. Also, from Equation (8), $\text{Cov}\{f(X), d_o(X)\} \geq 0$ is a necessary and sufficient condition for $\mathbb{E}\{f(Y)\} \geq \mathbb{E}\{f(X)\}$. Hence, conditions specified in Equation (12) and Eq. (13) collectively ensure $B_{\text{local}} \geq 0$.

Cases where B_{global} and B_{local} disagree (difference of the signs): Note that,

$$\begin{aligned}
B_{\text{local}} > 0 &\iff \mathbb{E}\{q_f(X)\} > \mathbb{E}\{f(X)\} \\
&\iff \bar{d}\mathbb{E}\Big\{f(U)\mathcal{A}(V)\Big|(U,V) \sim \text{Uniform}(E)\Big\} > \mathbb{E}\{f(X)\} \\
&\iff \mathbb{E}\Big\{f(U)\mathcal{A}(V)\Big|(U,V) \sim \text{Uniform}(E)\Big\} > \frac{1}{\bar{d}}\mathbb{E}\{f(X)\} \\
&\iff \mathbb{E}\Big\{f(U)\mathcal{A}(V)\Big|(U,V) \sim \text{Uniform}(E)\Big\} > \mathbb{E}\{\mathcal{A}(V)\}\mathbb{E}\{f(X)\} \\
&\qquad \text{(by noting that } \mathbb{E}\{\mathcal{A}(V)\} = \frac{1}{\bar{d}}) \\
&\iff \text{Cov}\Big\{f(U)\mathcal{A}(V)\Big|(U,V) \sim \text{Uniform}(E)\Big\} > -\mathbb{E}\{\mathcal{A}(V)\}\big(\mathbb{E}\{f(Y)\} - \mathbb{E}\{f(X)\}\big) \\
&\qquad \text{(by subtracting } \mathbb{E}\{f(U)\mathbb{E}\{\mathcal{A}(V)\} \text{ from both sides)} \\
&\iff \text{Cov}\Big\{f(U)\mathcal{A}(V)\Big|(U,V) \sim \text{Uniform}(E)\Big\} > -\frac{1}{\bar{d}}B_{\text{global}}.
\end{aligned}$$

Hence, when $B_{\text{global}} < 0$, $B_{\text{local}} > 0$ if and only if $\text{Cov}\Big\{f(U)\mathcal{A}(V)\Big|(U,V) \sim \text{Uniform}(E)\Big\} > -\frac{1}{\bar{d}}B_{\text{global}} = \frac{|B_{\text{global}}|}{\bar{d}}$. Similarly, when $B_{\text{global}} > 0$, $B_{\text{local}} < 0$ if and only if $\text{Cov}\Big\{f(U)\mathcal{A}(V)\Big|(U,V) \sim \text{Uniform}(E)\Big\} < -\frac{1}{\bar{d}}|B_{\text{global}}|$ (where the absolute value is not really necessary but introduced only to compare the two cases easily). This proves the two cases considered in Sec. 2.2.3 of the main text.

Supplementary Note 2: Empirical Results

2.1 Individual-level Perception Bias

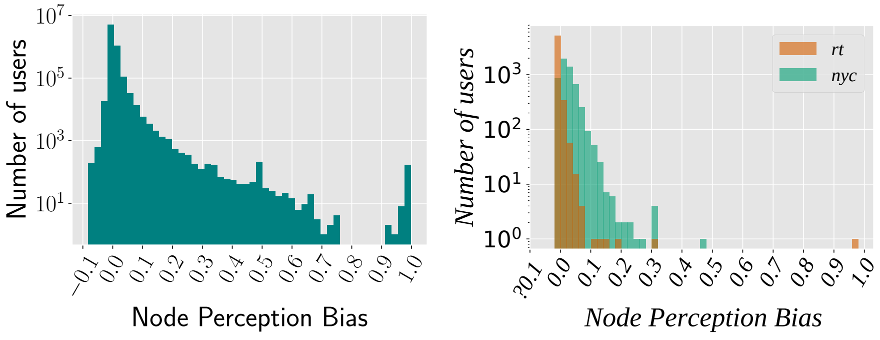

(a) (b)

Supplementary Figure 1: Individual-level perception bias $q_{f_h}(v) - \mathbb{E}\{f(X)\}$ for (a) all hashtags h and all nodes $v \in V$, and (b) for two hashtags with similar global prevalence, but with positive (#nyc) and negative (#rt) B_{local}. This illustrates that most hashtags are positively biased for individuals, with bias levels that do not depend on global prevalence.

Using Equation (9) we can compute *individual-level perception bias* for hashtag h as difference between perception of the *individual* about hashtag h and its global prevalence. $f_h(v)$ shows whether user v used hashtag h or not. The perception of node v about hashtag h can be shown as $q_{f_h}(v)$. Then the *individual-level perception bias* for hashtag h is:

$$B_h(v) = q_{f_h}(v) - \mathbb{E}\{f_h(X)\},$$

where $\mathbb{E}\{f_h(X)\}$ is the global prevalence of hashtag h. Supplementary Figure 1a shows the empirical distribution of $B_h(v)$ for all users and hashtags. Most of the mass of the histogram is for $B_h(v) > 0$, suggesting that most of the people in our data overestimate the popularity of these hashtags.

Supplementary Figure 1b compares individual-level perception bias for two hashtags that have similar global prevalence: #nyc ($\mathbb{E}\{f(X)\} = 0.021$) and #rt ($\mathbb{E}\{f(X)\} = 0.019$). Of the two hashtags, #nyc is perceived as more popular (with $B_{\text{local}_{\#nyc}} = 0.022$), but #rt appears less popular (with $B_{\text{local}_{\#rt}} = -0.011$) than it is globally.

2.2 Local and Global Bias

Global and local perception bias may either overestimate or underestimate the global prevalence of an attribute $\mathbb{E}\{f(X)\}$, depending on the values of the covariance between a node's attribute value and its out-degree and the attribute

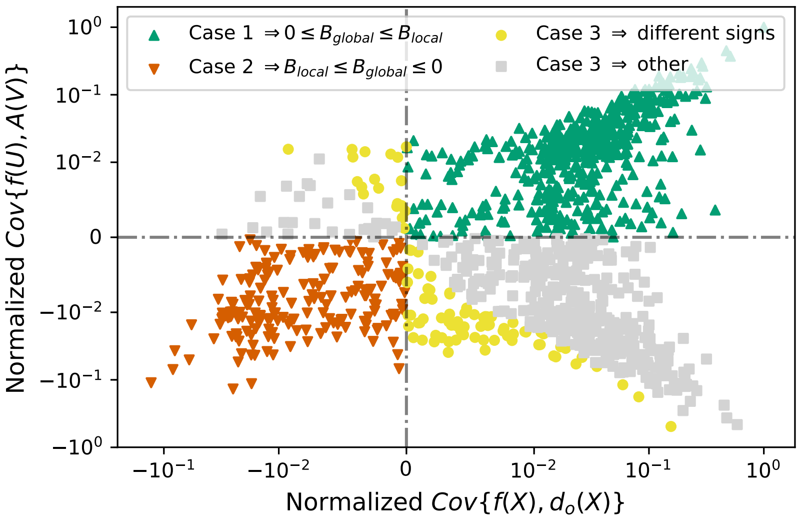

Case	Covariances	global/local bias	No. of hashtags
1	$\mathrm{Cov}\{f(U), \mathcal{A}(V) \mid (U,V) \sim \mathrm{Uniform}(E)\} \geq 0$<br>$\mathrm{Cov}\{f(X), d_o(X)\} \geq 0$	$0 \leq B_{\mathrm{global}} \leq B_{\mathrm{local}}$	474
2	$\mathrm{Cov}\{f(U), \mathcal{A}(V) \mid (U,V) \sim \mathrm{Uniform}(E)\} \leq 0$<br>$\mathrm{Cov}\{f(X), d_o(X)\} \leq 0$	$B_{\mathrm{local}} \leq B_{\mathrm{global}} \leq 0$	187
3	$\mathrm{Cov}\{f(U), \mathcal{A}(V) \mid (U,V) \sim \mathrm{Uniform}(E)\}$,<br>$\mathrm{Cov}\{f(X), d_o(X)\}$ have opposite signs	a) $B_{\mathrm{global}} \leq 0 \leq B_{\mathrm{local}}$<br>b) $B_{\mathrm{local}} \leq 0 \leq B_{\mathrm{global}}$<br>Other	19<br>75<br>398

Supplementary Figure 2: Value of $\mathrm{Cov}\{f(U), \mathcal{A}(V) \mid (U,V) \sim \mathrm{Uniform}(E)\}$ and $\mathrm{Cov}\{f(X), d_o(X)\}$ for all hashtags. Both variables are normalized by dividing to maximum value of the variable. The color represents the three cases. The table shows the number of hashtags that fall into each case.

value and attention along a random friend–follower link. We enumerate the cases below (proofs on Section 1.2).

Case 1 : $\text{Cov}\{f(U), \mathcal{A}(V)|(U, V) \sim \text{Uniform}(E)\} \geq 0$ and $\text{Cov}\{f(X), d_o(X)\} \geq 0$.
In this case, B_{global} and B_{local} both overestimate $\mathbb{E}\{f(X)\}$, and local bias is larger than global bias, i.e $B_{\text{local}} \geq B_{\text{global}} \geq 0$.

Case 2 : $\text{Cov}\{f(U), \mathcal{A}(V)|(U, V) \sim \text{Uniform}(E)\} \leq 0$ and $\text{Cov}\{f(X), d_o(X)\} \leq 0$.
In this case, B_{global} and B_{local} both underestimate $\mathbb{E}\{f(X)\}$, and local bias is smaller than global bias, i.e $B_{\text{local}} \leq B_{\text{global}} \leq 0$.

Case 3 : $\text{Cov}\{f(U), \mathcal{A}(V)|(U, V) \sim \text{Uniform}(E)\}$ and $\text{Cov}\{f(X), d_o(X)\}$ have opposite signs.
In this case the signs of the B_{global} and B_{local} can be different, with one overestimating and the underestimating the global prevalence of the attribute. These extreme cases are caused by the large covariance between the attribute and attention along a random friend–follower link. We make this case more precise with the following results:

(a) If $B_{\text{global}} < 0$, then $B_{\text{local}} > 0$ if and only if
$\text{Cov}\{f(U), \mathcal{A}(V)|(U, V) \sim \text{Uniform}(E)\} > \frac{|B_{\text{global}}|}{d}$

(b) If $B_{\text{global}} > 0$, then $B_{\text{local}} < 0$ if and only if
$\text{Cov}\{f(U), \mathcal{A}(V)|(U, V) \sim \text{Uniform}(E)\} < \frac{-|B_{\text{global}}|}{d}$.

We separate the hashtags in our Twitter data sample into the above three cases (Supplementary Figure 2). The majority of hashtags fall into cases 1 and 2, suggesting that local perception bias is larger in magnitude than the global perception bias.

Case 1: The hashtags (shown in green in Supplementary Figure 2) are used by popular users who are followed by high attention followers. These hashtags include popular memes and political events, among others. Some examples of these hashtags are *#ferguson*, *#tbt*, *#icebucketchallenge*, *#mikebrown*, *#emmys*, *#tech*, *#nyc*, *#ebola*, *#robinwilliams*, *#sxsw*, *#alsicebucketchallenge*, *#applelive*, *#netneutrality*, *#worldcup*, *#startups*, *#michaelbrown*, *#earthquake*, *#apple*, *#sf*, *#iraq*. Interestingly, all hashtags listed as top 20 in Figure 3 belong to this case except *#social_media* and *#ff*.

Case 2: Some of the hashtags falling into this case (shown in red in Supplementary Figure 2) include *#rt*, *#tcot*, *#follow*, *#retweet*, *#oscars*, *#teamfollowback*, *#leadfromwithin*, *#mtvhottest*, *#teaparty*, *#shoutout*, *#pjnet*, *#cdnpoli*, *#gazaunderattack*, *#uniteblue*, *#asmsg*, *#tlot*, *#freepalestine*, *#ccot*, *#tfb*, *#np*. These hashtags are used by unpopular users; examples of these hashtags are the last-10 hashtags of Figure 3 except *#quote*.

Case 3: The hashtags falling into the left quadrant of Supplementary Figure 2 (in yellow) include *#sotu*, *#occupy*, *#marriageequality*, *#sandy*, *#haiyan*, *#esp*, *#openingday*, *#doma*, *#mex*, *#lightsout*, *#onthisday*, *#ufc*, *#ww1*, *#wimbledon*, *#oscar*, *#joinin*, *#9*, *#ukedchat*, *#uru*.

The hashtags falling into the right quadrant include *#quote*, *#quotes*, *#win*, *#news*, *#kindle*, *#author*, *#management*, *#p2*, *#romance*, *#mktg*, *#iartg*, *#leaders*, *#ww*, *#b*, *#so*, *#mystery*, *#children*, *#aine*, *#autism*, *#lp*.

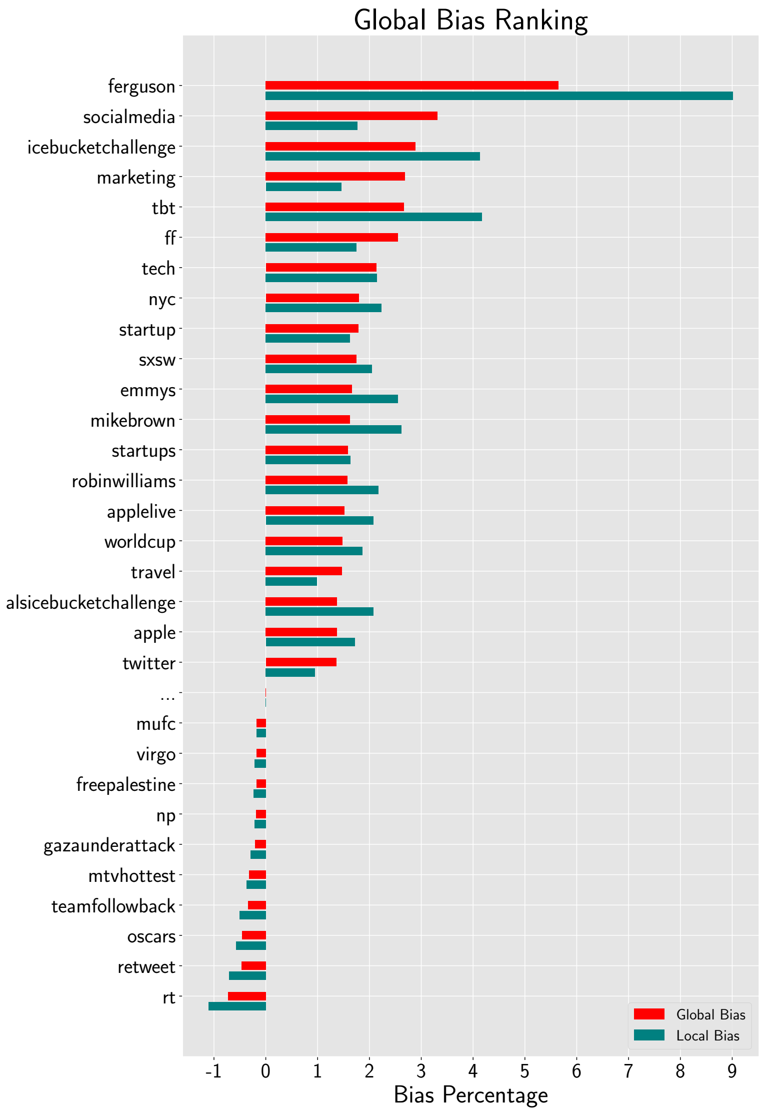

Supplementary Figure 3: The ranking of popular Twitter hashtags based on *Global bias*. Top-20 and bottom-10 are included in the ranking. The bars compare Global bias (B_{global}) and Local Bias (B_{local}).

2.3 Global Bias Ranking

Supplementary Figure 3 shows the ranking of popular Twitter hashtags based on global bias (B_{global}). Top-20 and bottom-10 are included in the ranking. There are 94 hashtags among 1153 with opposite sign of local bias and global bias, although both bias values for these hashtags are close to zero. Among the remaining hashtags, 661 (62%) have larger local bias than global bias, and 398 (38%) have larger global bias than local bias.

Supplementary Note 3: Polling Algorithm

3.1 Follower Perception Polling (FPP) Algorithm

The proposed polling algorithm samples random followers (step 1 of Algorithm 1) and asks about their perceptions (step 2 of Algorithm 1):
"What do you think is the fraction of individuals with attribute 1?"
We call the proposed algorithm Follower Perception Polling (FPP) algorithm.

Algorithm 1: Follower Perception Polling (FPP) Algorithm

Input: Graph $G = (V, E)$, perceptions $q_f : V \rightarrow \mathbb{R}^+$, sampling budget b.
Output: Estimate $\hat{\text{f}}_{\text{FPP}}$ of $\mathbb{E}\{f(X)\} = \frac{\sum_{v \in V} f(v)}{N}$.

1. Sample a set $S \subset V$ of b followers independently from the distribution

$$p_v = \frac{d_i(v)}{\sum_{v' \in V} d_i(v')}, \quad \forall v \in V.$$

2. Compute the estimate

$$\hat{\text{f}}_{\text{FPP}} = \frac{1}{b} \sum_{v \in S} q_f(v). \tag{18}$$

3.2 Bias of the Polling Estimate

Theorem 3. *The bias of the estimate $\hat{\text{f}}_{\text{FPP}}$ computed in Algorithm 1 is equal to the global perception bias B_{global} i.e.*

$$\text{Bias}(\hat{\text{f}}_{\text{FPP}}) = \mathbb{E}\{\hat{\text{f}}_{\text{FPP}}\} - \mathbb{E}\{f(X)\} \tag{19}$$

$$= B_{global} \tag{20}$$

Proof. Let e_v denote the $n \times 1$ dimensional unit vector with 1 at the vth element and zeros elsewhere. Then,

$$q_f(v) = e_v^T D_i^{-1} A^T f \tag{21}$$

and let $M = \sum_{v \in V} d_i(v)$. With Z defined in Equation (3) of the main text,

$$\mathbb{E}\{\hat{\mathrm{f}}_{\mathrm{FPP}}\} = \mathbb{E}\{q_f(Z)\} = \sum_{v \in V} \frac{d_i(v)}{M} q_f(v) \tag{22}$$

$$= \sum_{v \in V} \frac{d_i(v)}{M} \left(e_v^T D_i^{-1} A^T f \right) = \frac{1}{M} \mathbb{1}^T D_i D_i^{-1} A^T f \tag{23}$$

$$= \frac{1}{M} \mathbb{1}^T A^T f \tag{24}$$

$$= \sum_{v \in V} f(v) \frac{d_o(v)}{M} = \mathbb{E}\{f(Y)\} \tag{25}$$

Therefore,

$$\mathrm{Bias}\{\hat{\mathrm{f}}_{\mathrm{FPP}}\} = \mathbb{E}\{\hat{\mathrm{f}}_{\mathrm{FPP}}\} - \mathbb{E}\{f(X)\} \tag{26}$$

$$= \mathbb{E}\{f(Y)\} - \mathbb{E}\{f(X)\} = B_{\text{global}} \tag{27}$$

□

3.3 Variance of the Polling Estimate

Theorem 4. *Consider the estimate* $\hat{\mathrm{f}}_{\mathrm{FPP}}$ *generated by Algorithm 1 for a graph* $G = (V, E)$ *with labels* $f : V \to \{0, 1\}$. *If the degree-discounted bibliographic coupling matrix* B_d *is connected, non-bipartite, then*

$$\mathrm{Var}(\hat{\mathrm{f}}_{\mathrm{FPP}}) = \frac{f^T D_o^{1/2}}{bM} \left(D_o^{-1/2} A D_i^{-1} A^T D_o^{-1/2} - \frac{D_o^{1/2} \mathbb{1}\mathbb{1}^T D_o^{1/2}}{M} \right) D_o^{1/2} f \tag{28}$$

$$\leq \frac{1}{bM} \lambda_2 ||D_o^{1/2} f||^2 \tag{29}$$

where, $M = \sum_{v \in V} d_i(v)$, λ_2 *is the second largest eigenvalue of* B_d, f *is the* $N \times 1$ *dimensional vector of binary attributes.*

Proof. Since $\hat{\mathrm{f}}_{\mathrm{FPP}}$ is the average of the perceptions of b independently sampled random followers,

$$\mathrm{Var}(\hat{\mathrm{f}}_{\mathrm{FPP}}) = \frac{1}{b} \mathrm{Var}(q_f(Z)) = \frac{1}{b} \left(\mathbb{E}\{q_f^2(Z)\} - \mathbb{E}\{q_f(Z)\}^2 \right) \tag{30}$$

where, Z is a random follower. Consider $\mathbb{E}\{q_f^2(Z)\}$.

$$\mathbb{E}\{q_f^2(Z)\} = \sum_{v \in V} \frac{d_i(v)}{M} q_f^2(v) = \sum_{v \in V} \frac{d_i(v)}{M} f^T A D_i^{-1} e_v e_v^T D_i^{-1} A^T f \tag{31}$$

(by substituting for $q_f(v)$ from Equation (21))

$$= \frac{1}{M} \left(f^T A D_i^{-1} \Big(\sum_{v \in V} d_i(v) e_v e_v^T \Big) D_i^{-1} A^T f \right) \tag{32}$$

$$= \frac{1}{M} f^T A D_i^{-1} A^T f \tag{33}$$

Hence,

$$\begin{aligned}
\mathrm{Var}(q_f(Z)) &= \mathbb{E}\{q_f^2(Z)\} - \mathbb{E}\{q_f(Z)\}^2 && (34)\\
&= \frac{1}{M} f^T A D_i^{-1} A^T f - \frac{1}{M^2} f^T A \mathbb{1}\mathbb{1}^T A^T f && (35)\\
&\quad \text{(by substituting from Equations (24) and (33))}\\
&= \frac{1}{M} f^T \left(A D_i^{-1} A^T - \frac{1}{M} A \mathbb{1}\mathbb{1}^T A^T \right) f && (36)\\
&= \frac{f^T D_o^{1/2}}{M} \left(D_o^{-1/2} A D_i^{-1} A^T D_o^{-1/2} - \frac{D_o^{1/2}\mathbb{1}\mathbb{1}^T D_o^{1/2}}{M} \right) D_o^{1/2} f && (37)\\
&\leq \frac{||D_o^{1/2} f||^2}{M} \left|\left| D_o^{-1/2} A D_i^{-1} A^T D_o^{-1/2} - \frac{D_o^{1/2}\mathbb{1}\mathbb{1}^T D_o^{1/2}}{M} \right|\right| && (38)
\end{aligned}$$

where, for a matrix A, $||A||$ denotes the spectral norm (largest singular value) and Equation (38) is obtained by applying the Cauchy-Schwarz inequality.

Note that the degree-discounted bibliographic coupling-matrix,

$$B_d = D_o^{-1/2} A D_i^{-1} A^T D_o^{-1/2} = \left(D_o^{-1/2} A D_i^{-1/2}\right)\left(D_o^{-1/2} A D_i^{-1/2}\right)^T$$

is a symmetric, positive semi-definite matrix. Hence, all eigenvalues are non-negative. Further, $\frac{D_o^{1/2}\mathbb{1}}{\sqrt{M}}$ is the eigenvector with all non-negative elements and corresponds to eigenvalue 1. Hence,

$$\left|\left| D_o^{-1/2} A D_i^{-1} A^T D_o^{-1/2} - \frac{D_o^{1/2}\mathbb{1}\mathbb{1}^T D_o^{1/2}}{M} \right|\right| = \lambda_2$$

where, λ_2 is the second largest eigenvalue of B_d. Then, the result follows from (Equation 30). □

3.4 Heuristic Follower Perception Polling

It may not always be feasible to sample followers at random, specifically, by sampling nodes proportional to their in-degree. Our *Follower Perception Polling* (FPP) algorithm samples nodes based on their in-degree. For computing in-degree we need to access whole network; however, in many cases the whole network may not be available. We can approximate the sampling used by FPP algorithm using the following heuristic. Instead of sampling b nodes weighted based on their in-degree (which needs information about whole network), the proposed Approximate-FPP algorithm samples b nodes at random, and as a second step, it samples b nodes from followers of these nodes. This procedure does not need whole network structure, and it could be shown that it is an approximation of the FPP algorithm. Figure 4d shows the performance of Approximate-FPP in comparison to the other polling algorithms.

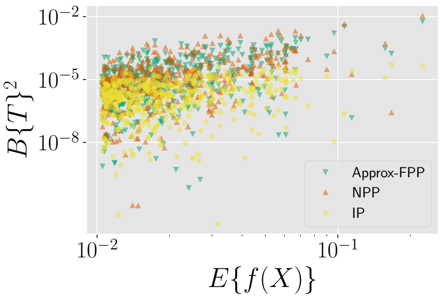

(a)

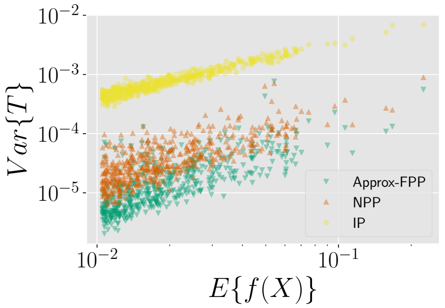

(b)

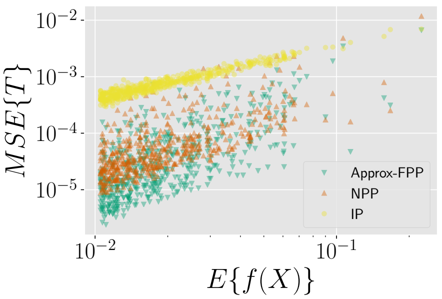

(c)

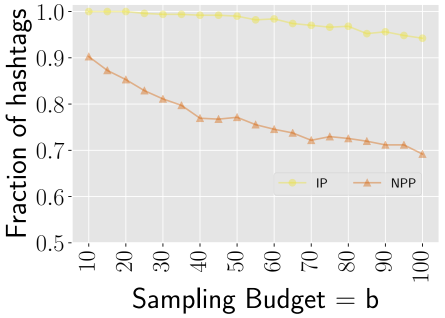

(d)

Supplementary Figure 4: Comparison of polling algorithms for estimating the global prevalence of Twitter hashtags. Variation of (a) squared bias ($\text{Bias}\{T\}^2$), (b) variance ($\text{Var}\{T\}$) and (c) mean squared error ($\text{Bias}\{T\}^2 + \text{Var}\{T\}$) of the polling estimate (*IP*, *NPP* and *Approximated-FPP* as T - polling algorithm -) as a function of a hashtag's global prevalence $\mathbb{E}\{f(X)\}$. Each point represents a different hashtag and a fixed sampling budget $b = 25$. (d) Fraction of hashtags where the proposed FPP algorithm with the sampling heuristic (Approximate-FPP) outperforms the other polling methods in terms of MSE. The fraction for NPP approaches 0.5, and for IP approaches 0.8 as sampling budget b increases. The main difference between Approximate-FPP and FPP is in Figure (d) with low amount of budget b. In this case, the ratio of hashtags where Approximate-FPP could perform better than NPP is around 0.8 compare with 0.9 for FPP algorithm.
